# Human Papillomavirus in Bowen Disease: Site-Specific Prevalence, Genotype Distribution, and Clinical Implications Across Nail Apparatus, Cutaneous, and Anogenital Sites

**DOI:** 10.3390/ijms27083555

**Published:** 2026-04-16

**Authors:** Emi Dika, Carlotta Baraldi, Federico Venturi, Aurora Maria Alessandrini, Sabina Vaccari, Simona Venturoli, Gabriele Argenziano, Tiziano Ferrari, Tiziana Lazzarotto, Elisabetta Magnaterra

**Affiliations:** 1 Department of Medical and Surgical Sciences (DIMEC), University of Bologna, 40138 Bologna, Italy; 2 Oncologic Dermatology Unit, IRCCS Azienda Ospedaliero-Universitaria di Bologna, 40138 Bologna, Italy; 3 Microbiology Unit, IRCCS Azienda Ospedaliero-Universitaria di Bologna, 40138 Bologna, Italy; 4 Department of Medical and Surgical Sciences, Section of Microbiology, University of Bologna, 40138 Bologna, Italy

**Keywords:** Bowen disease, squamous cell carcinoma, human papillomavirus, HPV genotypes

## Abstract

Bowen disease (BD), or squamous cell carcinoma (SCC) in situ, represents a histologically defined but biologically heterogeneous group of intraepithelial neoplasms arising across different epithelial compartments. Human papillomavirus (HPV) plays a well-established causal role in anogenital squamous intraepithelial neoplasia, whereas its contribution to extragenital BD, including nail apparatus and general cutaneous lesions, has remained controversial. We performed a narrative review of the literature to synthesize current evidence on HPV prevalence, genotype distribution, and pathogenetic relevance in BD across three anatomical sites: nail apparatus, general cutaneous skin, and anogenital region. Available data reveal a clear site-dependent gradient of HPV involvement. Anogenital BD is overwhelmingly driven by high-risk α-HPV genotypes and shares molecular hallmarks of HPV-mediated carcinogenesis. Nail apparatus BD shows a consistently high prevalence of transforming α-HPV types, suggesting a biologically distinct subset of extragenital disease. In contrast, general cutaneous BD demonstrates highly variable HPV detection, predominantly involving β- and occasionally γ-HPV types, with evidence supporting a permissive or incidental rather than causal role. These findings indicate that BD should not be regarded as a unified viral neoplasm but as a convergent histologic phenotype arising from distinct pathogenetic pathways. Anatomical context is therefore essential for interpreting HPV detection and its diagnostic and clinical implications.

## 1. Introduction

BD, or SCC in situ, is defined histologically by full-thickness epidermal dysplasia with preservation of the basement membrane [1,2,3]. Since its original description by John Templeton Bowen in 1912, BD has been recognized as a slowly progressive intraepithelial malignancy with the potential for invasive transformation [1]. Clinically, BD presents as a persistent erythematous, scaly, or hyperkeratotic plaque that may arise on sun-exposed or sun-protected skin, as well as within mucosal and anogenital regions [1,2,3]. Although traditionally unified under a single histopathologic diagnosis, BD is increasingly understood as a heterogeneous condition with site-specific etiologic drivers and divergent molecular pathways [1,2,3].

**Figure 1:**
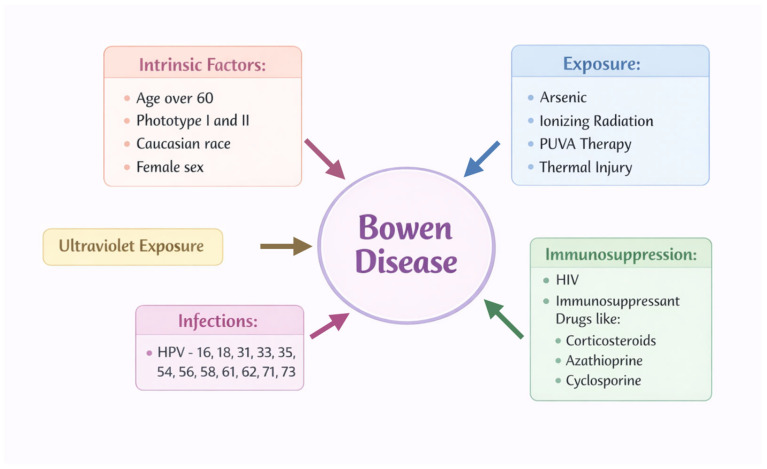
Schematic overview of the main risk factors associated with Bowen disease.

Among the established risk factors for BD are ultraviolet (UV) radiation, immunosuppression, arsenic exposure, chronic inflammation, trauma, and viral oncogenesis (Figure 1) [3,4,5].

Of these, HPV occupies a uniquely paradoxical position. Its causal role in anogenital squamous intraepithelial neoplasia is unequivocal, supported by decades of molecular, epidemiologic, and interventional evidence, including the dramatic reduction in cervical and other anogenital precancers following prophylactic HPV vaccination [6,7,8]. In contrast, its contribution to extragenital keratinocyte tumors—including BD of the skin and nail apparatus—has remained controversial for more than three decades [9,10,11,12].

**Figure 2:**
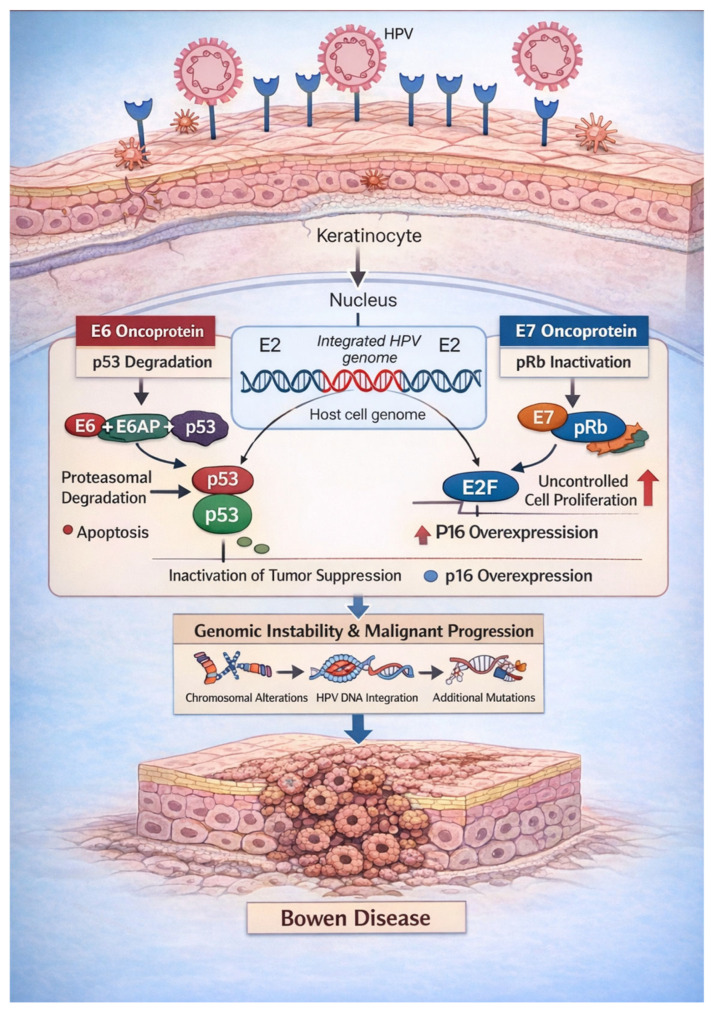
Mechanistic role of HPV in the pathogenesis of Bowen disease.

HPVs are small, non-enveloped, double-stranded DNA viruses with a marked tropism for epithelial keratinocytes of the skin and mucous membranes. Among more than 200 identified genotypes, high-risk α-HPVs—most notably HPV-16 and HPV-18—have been conclusively implicated in epithelial carcinogenesis, particularly in anogenital and oropharyngeal sites [6,8,13]. Oncogenic transformation is primarily mediated by the viral E6 and E7 oncoproteins, which disrupt key cell-cycle regulatory pathways through functional inactivation of the tumor suppressors p53 and retinoblastoma protein (pRb), leading to genomic instability and uncontrolled cellular proliferation (Figure 2) [14,15]. Persistent infection and, in many cases, integration of viral DNA into the host genome further amplify E6 and E7 expression and are strongly associated with malignant progression [16,17,18].

HPV infects keratinocytes and may integrate into the host genome, often with disruption of the viral E2 gene, leading to uncontrolled expression of the viral oncoproteins E6 and E7. E6 promotes ubiquitin-mediated degradation of p53 via interaction with E6-associated protein (E6AP), impairing DNA damage response, cell cycle arrest, and apoptosis. In parallel, E7 inactivates pRb, resulting in release of E2F transcription factors, cell cycle progression, and compensatory upregulation of p16INK4a. The combined effects of E6 and E7 drive uncontrolled keratinocyte proliferation, genomic instability, and accumulation of genetic alterations. These molecular events contribute to the development of BD.

In contrast, cutaneous HPVs belonging to the β- and γ-genera are ubiquitous components of the normal skin virome and are frequently detected in healthy individuals [11]. Experimental and epidemiologic data suggest that these viruses lack consistent transforming activity and may instead act as permissive cofactors in carcinogenesis, particularly in the context of UV-induced DNA damage and impaired immune surveillance [19]. This virologic heterogeneity may partly explain the site-dependent and often conflicting associations between HPV and BD.

Early reports in the 1980s and 1990s identifying mucosal high-risk HPV DNA in digital and extragenital BD lesions raised the provocative hypothesis that HPV might act as a broader oncogenic driver beyond mucosal epithelia [20]. Subsequent studies, however, produced highly variable detection rates, ranging from negligible to more than 50%, depending on the population studied, the anatomical site examined, and the molecular methods employed [9,10,11]. These inconsistencies fueled persistent uncertainty about whether HPV functions as a primary oncogenic driver, a permissive cofactor, or merely an incidental commensal in cutaneous lesions [10,11].

More recently, a coherent site-dependent pattern has begun to emerge. Nail apparatus BD demonstrates a consistently high prevalence of HPV, dominated by high-risk α-HPV genotypes, particularly HPV-16 [21,22,23,24]. General cutaneous BD, by contrast, exhibits inconsistent and method-dependent HPV detection, often enriched for β-HPV types with uncertain oncogenic relevance [11,25,26]. Anogenital BD remains overwhelmingly HPV-driven, mirroring the molecular and clinical behavior of other mucosal squamous intraepithelial neoplasias [6,27].

Importantly, the term “Bowen disease” encompasses a biologically heterogeneous group of SCC in situ lesions arising across different epithelial compartments. Within the anogenital region, several site-specific entities are traditionally distinguished, including erythroplasia of Queyrat (EQ) of the glans penis and prepuce, vulvar intraepithelial neoplasia (VIN), penile intraepithelial neoplasia (PeIN), and bowenoid papulosis [28,29,30,31,32,33]. Although these lesions share the histologic hallmark of full-thickness epithelial dysplasia, they differ in clinical presentation, terminology, and molecular underpinnings [28,29,30,31,32,33]. EQ and differentiated forms of VIN and PeIN may arise independently of HPV and are often associated with chronic inflammatory dermatoses or lichen sclerosus, whereas usual-type VIN, PeIN, and bowenoid papulosis are strongly associated with high-risk α-HPV infection, particularly HPV-16 [33,34]. Modern classification systems therefore distinguish HPV-related and HPV-independent pathways of anogenital squamous carcinogenesis, reflecting fundamental differences in pathogenesis, risk of progression, and clinical behavior [35].

This pronounced anatomical and biological gradient strongly suggests that BD should not be regarded as a unified viral neoplasm, but rather as a convergent histologic phenotype arising from distinct pathogenetic pathways [27,33,34]. Recognition of this heterogeneity has direct implications for diagnosis, mechanistic understanding, and emerging translational strategies, including noninvasive viral detection and HPV-targeted therapies.

Accordingly, this review synthesizes the existing literature on HPV prevalence, genotype distribution, and pathogenetic relevance in BD across three major anatomical compartments—nail apparatus, general cutaneous skin, and anogenital region—and critically examines the biological plausibility and clinical implications of this heterogeneity.

## 2. Methods

A comprehensive literature search was conducted in PubMed/MEDLINE, Embase, and Scopus from database inception through January 2026. Search terms included combinations of: BD, squamous cell carcinoma in situ, human papillomavirus, HPV, nail unit, ungual, periungual, cutaneous, extragenital, anogenital, genital, and p16. Reference lists of relevant articles and reviews were manually screened to identify additional studies.

Eligible studies included original research articles, retrospective case series, molecular pathology studies, and systematic reviews reporting HPV detection in histologically confirmed BD or SCC in situ. Studies were included if they provided extractable data on HPV prevalence, genotype distribution, or detection methodology. Case reports were included selectively when they contributed novel mechanistic or diagnostic insights, particularly for nail apparatus disease. Studies were excluded if they lacked clear histopathologic confirmation of BD, failed to specify anatomical site, or relied exclusively on serology without tissue-based HPV detection. For each study, the following data were extracted: anatomical site (nail apparatus, general cutaneous, or anogenital), sample size, population characteristics (age, sex, immunosuppression status), specimen type (fresh tissue, formalin-fixed paraffin-embedded tissue, or surface swab), HPV detection method (PCR primer sets, in situ hybridization, immunohistochemistry), HPV positivity rate, genotype distribution (α-, β-, γ-HPV), and use of surrogate markers such as p16INK4A.

A narrative synthesis was performed, stratifying findings by anatomical site. HPV prevalence and genotype distribution were compared across nail apparatus, general cutaneous, and anogenital BD. Particular attention was given to methodological drivers of variability, including primer selection, specimen type, and contamination control. Where possible, detection rates were contextualized within their methodological and population-specific frameworks. Given the heterogeneity of study designs and detection methods, formal meta-analysis was not performed. Methodological rigor was qualitatively assessed based on tissue specificity of sampling, breadth of HPV primer coverage, contamination controls, and clarity of anatomical classification.

## 3. HPV in Bowen Disease Across Anatomical Sites

### Nail Apparatus Bowen Disease

Among extragenital anatomical compartments, nail apparatus BD shows the strongest and most consistent association with HPV. Retrospective series and molecular studies repeatedly report HPV detection in approximately 60–80% of nail unit BD and SCC cases [9,23,36,37,38]. Published cases and case series reporting HPV detection in nail unit Bowen disease are summarized in Table 1.

In a 12-case retrospective series, Perruchoud and colleagues identified HPV DNA in 75% of nail BD lesions using polymerase chain reaction, with HPV-16 as the predominant genotype, followed by HPV-73 and HPV-52 [36]. Larger narrative syntheses and systematic reviews further estimate that HPV-16 accounts for approximately half of in situ nail SCCs and nearly three-quarters of invasive nail SCCs, with additional α-HPV genotypes—including HPV-31, -33, -35, -52, -58, -67, and -73—also implicated [9,11,23].

This predominance of high-risk α-HPVs in nail apparatus BD contrasts sharply with the genotype distribution observed in extragenital cutaneous BD and is consistent with a pathogenetic pathway that may resemble mucosal squamous neoplasia more than UV-driven keratinocyte tumors [9,25].

Several anatomical and clinical features have been proposed to support this association. The periungual and hyponychial epithelium is thin, poorly keratinized, frequently traumatized, and particularly susceptible to viral inoculation [9,39,40]. Chronic microtrauma, onycholysis, and paronychia may facilitate viral entry and persistence [41]. Unlike general cutaneous skin, the nail apparatus is largely shielded from UV radiation, reducing the dominance of UV-driven mutagenesis and potentially amplifying the relative importance of viral oncogenesis [9,37].

Genitodigital transmission provides an additional mechanistic framework. HPV DNA has been detected in fingernails and periungual skin of individuals with active genital HPV infection, and concordant HPV genotypes have been identified in paired genital and digital lesions in a subset of patients [20,42,43,44]. In several cohorts, genital HPV-related disease preceded nail unit SCC by more than a decade, consistent with long-term viral persistence and delayed malignant transformation [9,23].

Recent molecular and case-based evidence further reinforces the biological distinctiveness of nail apparatus BD. Ochiai and colleagues demonstrated detection of high-risk HPV-58 both in formalin-fixed tissue and in preoperative surface swab samples obtained from keratotic nail folds, highlighting the feasibility of noninvasive HPV detection as a diagnostic adjunct [22].

More recently, Guerrero-González and colleagues reported multiple periungual pigmented Bowen lesions in an immunocompetent 13-year-old boy, with HPV-16 detected by PCR in all three lesions. Histopathology revealed koilocytosis, and a concomitant genital wart was observed. All lesions occurred on the same hand and were associated with longitudinal melanonychia [21]. This case is notable for several reasons: it demonstrates that HPV-driven nail BD is not confined to older adults; it reinforces the predominance of high-risk α-HPV genotypes in ungual disease; and it provides compelling clinical support for genitodigital transmission as a biologically plausible pathway.

Clinically, HPV-associated nail BD often presents as verrucous periungual or subungual lesions, longitudinal melanonychia, erythronychia, onycholysis, or nail dystrophy [9]. These nonspecific manifestations frequently lead to misdiagnosis as viral warts, onychomycosis, or traumatic nail disorders, resulting in diagnostic delays exceeding five years in some series [23,36,37]. The convergence of molecular, epidemiologic, and clinicopathologic data positions nail apparatus BD as a true HPV-associated intraepithelial neoplasm in a substantial proportion of cases.

**Table 1:** Summary of reported cases of HPV-associated Bowen disease of the nail unit. The table summarizes available evidence on HPV detection in nail unit Bowen disease, including study design, number of cases, HPV prevalence, genotype distribution, and key clinical or pathogenetic insights.

Reference	Study Design	No. of Cases	HPV Detection Rate	HPV Types Identified	Key Findings
Perruchoud et al., 2016 [36]	Retrospective series	12 cases (10 patients)	75%	Predominantly high-risk α-HPV (HPV-16 most common; others detected)	High prevalence of HPV in nail BD; long diagnostic delay; frequent verrucous presentation
Ochiai et al., 2024 [22]	Case report	1	Positive	HPV-58 (high-risk α-HPV)	Demonstrates utility of surface brush cytology for non-invasive HPV detection
Guerrero-González et al., 2023 [21]	Case report	1 (multiple lesions)	Positive	HPV-16	Multiple periungual BD lesions in pediatric patient; supports HPV-driven pathogenesis
Guldbakke et al., 2008 [45]	Case report	1	Positive	HPV-73	Associated with primary and recurrent periungual SCC in situ
Kreuter et al., 2009 [39]	Case series	6 periungual SCC/SCC in situ	~100% (periungual subset)	HPV-26, 33, 51, 56, 73 (not only HPV-16)	Strong association of periungual lesions with α-HPV; broader genotype spectrum
Ekeowa-Anderson et al., 2007 [43]	Case report	1	Positive	HPV-34 (α), HPV-21 (β)	Concordance between genital and periungual lesions; supports genitodigital transmission
Guitart et al., 1990 [40]	Case series	12 SCC (subset BD)	HPV detected in subset	Not fully typed	Suggests HPV as possible etiologic factor in nail SCC/BD
Nordin et al., 1994 [44]	Case report	1	Positive	HPV-16	Same HPV type detected in genital and digital BD; supports transmission link
Rüdlinger et al., 1989 [42]	Case report	1	Positive	HPV-35	HPV detected in both anogenital and periungual lesions; suggests autoinoculation
Hama et al., 2006 [20]	Case series	21 BD (mixed sites)	4.8% overall	HPV-31 (in BD case)	Low detection rate in mixed cutaneous BD; highlights site variability

## 4. General Cutaneous (Extragenital) Bowen Disease

In contrast to nail apparatus lesions, HPV prevalence in general cutaneous BD is highly variable, with reported detection rates ranging from less than 10% to over 50%, depending on the population studied and the molecular methods employed [11,25,26,46]. This variability reflects substantial heterogeneity in study design, primer selection, specimen type, tissue sampling depth, and population characteristics [11,25]. Studies investigating HPV detection in extragenital/extraungual Bowen disease are summarized in Table 2.

A systematic review of keratinocyte tumors reported HPV positivity in approximately 56% of BD lesions for β-HPV and 39% for α-HPV, although these aggregate estimates masked marked inter-study heterogeneity and wide confidence intervals [11]. In the same analysis, γ-HPV genotypes were detected in a smaller subset of BD lesions, accounting for approximately 5% of HPV-positive cases [11]. Molecular analyses of extragenital BD similarly demonstrate inconsistent detection, with mixed distributions of α-, β-, and, less frequently, γ-HPV genera, and strong dependence on detection methodology [25,26].

Unlike nail apparatus BD, general cutaneous BD is characterized by a predominance of β-HPV genotypes [11,26,47]. These viruses are ubiquitous components of the normal cutaneous virome and are frequently detected in healthy individuals, hair follicles, and sun-exposed skin, complicating causal inference. The detection of β-HPVs in BD lesions has been interpreted as reflecting commensal colonization or field cancerization rather than true oncogenic involvement [11].

Mechanistic models propose that β-HPVs act as permissive cofactors rather than direct oncogenic drivers. Experimental and translational studies suggest that β-HPV oncoproteins may impair DNA damage repair, apoptosis, and immune surveillance, thereby lowering the threshold for UV-induced carcinogenesis without directly sustaining malignant transformation [48,49]. The so-called “hit-and-run” hypothesis has been proposed, suggesting that β-HPVs may initiate early carcinogenic events but are not required for tumor maintenance [48,49].

Consistent with this model, p16 overexpression—a hallmark of oncogenic α-HPV activity—does not reliably correlate with HPV positivity in extragenital BD. Several studies have demonstrated discordance between HPV DNA detection and p16 immunohistochemistry, underscoring fundamental biological differences from mucosal HPV-driven disease [25,26].

A minority of extragenital BD lesions harbor α-HPV genotypes, particularly in digital or distal extremity lesions [20,25]. These cases likely reflect genitodigital transmission rather than a generalized cutaneous oncogenic pathway and may represent a biologically distinct subset closer to nail apparatus disease [20,25].

From a clinical standpoint, the inconsistent and method-dependent HPV prevalence in general cutaneous BD limits the utility of routine viral testing. Unlike nail apparatus and anogenital disease, HPV status in extragenital lesions does not reliably correlate with clinical phenotype, prognosis, or therapeutic response [25,26]. At present, HPV detection in cutaneous BD remains primarily of pathogenetic interest rather than direct clinical relevance.

**Table 2:** Summary of studies investigating HPV detection in extragenital/extraungual Bowen disease.

Reference	Study Design	No. of bd Cases	HPV Detection Rate	HPV Types Identified	Key Findings
Kim et al., 2024 [46]	Retrospective study	109	19.3% overall (pelvic 69%, digital 50%)	α-HPV (HPV-16 most common)	Higher prevalence in pelvic/digital regions; HPV associated with p53 negativity; no correlation with p16
Svajdler et al., 2016 [26]	Retrospective study (immunocompromised)	29	40%	Predominantly β-HPV; minority α-HPV	β-HPV predominance; p16 not a reliable marker of HPV infection
Neagu et al., 2022 [11]	Systematic review	290	~55.8% β, ~39% α	β > α >> γ	High heterogeneity; detection influenced by methodology
Conforti et al., 2024 [25]	Review/analysis	Multiple studies	Variable (~0–50%)	α, β, γ HPV	HPV likely acts as a cofactor rather than a primary oncogenic driver
Brunet-possenti et al., 2025 [47]	Retrospective study (cSCC incl. BD)	30 BD (subset)	HPV detected in 56.6% overall (α-HPV ~19%)	α-HPV (HPV-16 predominant)	Evidence of viral integration in subset; oncogenic role suggested but not definitive
Mitsuishi et al., 2003 [50]	Retrospective study	62	65%	Multiple α and β HPV types	High HPV prevalence but no difference in proliferation markers between HPV-positive and negative lesions
Lellis et al., 2017 [51]	Case report	1	Positive	High-risk α-HPV (16/18/31/33/35/39)	Pigmented Bowen disease of the finger; HPV-associated lesion mimicking melanoma

## 5. Anogenital Bowen Disease

Anogenital BD represents the prototypical HPV-driven intraepithelial neoplasm and constitutes the clearest example of virally mediated squamous carcinogenesis within the spectrum of SCC in situ. High-risk α-HPV genotypes—most prominently HPV-16 and HPV-18—are detected in the vast majority of lesions, with reported prevalence rates exceeding 90% in most series [6,31]. These lesions form part of a continuous pathogenetic and clinical spectrum encompassing usual-type VIN, PeIN, bowenoid papulosis, EQ, and high-grade anal intraepithelial neoplasia, all of which share a common viral etiology and a well-established risk of progression to invasive SCC [27,28,30,31,32,33,52,53].

At the molecular level, anogenital BD exhibits the canonical hallmarks of oncogenic HPV-driven transformation. Viral genome integration into host DNA leads to deregulated expression of the E6 and E7 oncoproteins, with subsequent functional inactivation of the p53 and pRb tumor suppressor pathways [8,13]. This process results in diffuse overexpression of p16INK4A, which serves as a highly sensitive and specific surrogate marker of transforming high-risk HPV infection and is now routinely incorporated into diagnostic algorithms for anogenital squamous intraepithelial lesions [8,54].

Contemporary classification systems further distinguish two major pathogenetic pathways of anogenital squamous carcinogenesis: an HPV-related pathway, encompassing usual-type VIN and PeIN and bowenoid papulosis, and an HPV-independent pathway, which includes differentiated VIN, differentiated PeIN, and EQ arising in the setting of chronic inflammatory dermatoses, particularly lichen sclerosus [27,33,52]. These pathways differ fundamentally in molecular drivers, age distribution, risk of progression, and clinical behavior, with HPV-related lesions typically affecting younger patients and demonstrating strong p16 expression, whereas HPV-independent lesions arise in older individuals, lack viral markers, and are frequently associated with TP53 mutations [27,29,33,52].

From a clinical standpoint, HPV status in anogenital BD has direct diagnostic, prognostic, and preventive relevance. p16 immunohistochemistry is widely used to confirm transforming HPV infection and to distinguish usual-type from differentiated precursor lesions [29,54]. Moreover, the central role of HPV in anogenital squamous intraepithelial neoplasia underlies the profound preventive impact of prophylactic HPV vaccination, which has been associated with a marked reduction in the incidence of high-grade cervical, vulvar, and anal intraepithelial neoplasia at the population level [7,55].

Within the broader context of BD, anogenital lesions therefore serve as a biological reference standard for HPV-driven intraepithelial carcinogenesis. Their well-defined viral etiology, reproducible molecular signature, and established clinical utility of viral biomarkers provide a benchmark against which the more heterogeneous and anatomically dependent HPV associations observed in nail apparatus and general cutaneous BD can be interpreted.

## 6. Comparative Synthesis

Collectively, the available evidence suggests an anatomical gradient in the strength, consistency, and biological relevance of HPV involvement in BD. At one extreme, anogenital SCC in situ represents a prototypical HPV-driven neoplasm, characterized by near-universal detection of high-risk α-HPV genotypes, reproducible molecular signatures of viral oncogenesis, and established clinical utility of viral biomarkers [6,10,13,27,31,54,55]. At the opposite end of the spectrum, general cutaneous BD exhibits inconsistent and method-dependent HPV detection, predominance of β-HPV genotypes with uncertain oncogenic relevance, and lack of reproducible correlation with molecular markers, clinical behavior, or therapeutic response [11,25,26]. Nail apparatus BD occupies an intermediate but biologically distinctive position, showing a consistently high prevalence of transforming α-HPV genotypes among extragenital sites and a pathogenetic profile more closely resembling mucosal squamous intraepithelial neoplasia than UV-driven keratinocyte tumors [11,23,36,37].

This site-dependent distribution supports the concept that BD does not represent a single virally mediated entity but rather a convergent histologic phenotype arising from fundamentally distinct carcinogenic pathways. In anogenital lesions, persistent high-risk HPV infection functions as a primary oncogenic driver [6,8,13]. In nail apparatus disease, viral oncogenesis appears to play a major but anatomically restricted role, likely facilitated by the unique epithelial microenvironment of the periungual unit and by genitodigital transmission [9,20,36]. In contrast, in general cutaneous BD, HPV—particularly β-HPV—appears more plausibly to act as a permissive cofactor in UV-induced carcinogenesis or to represent incidental colonization of a genetically altered field [11,25].

## 7. Methodological Drivers of Variability in HPV Detection

Interpretation of HPV prevalence across the BD spectrum is further complicated by substantial methodological heterogeneity among published studies. Detection rates are strongly influenced by primer selection and assay sensitivity, as broad-spectrum polymerase chain reaction systems identify a wider range of genotypes than type-specific assays, thereby altering both prevalence estimates and apparent genotype distributions [11,25,26]. Differences in tissue sampling depth also contribute to variability, as detection of viral DNA in perilesional epidermis, keratin debris, or hair follicles may inflate prevalence relative to analyses restricted to lesional epithelium [11,25].

Specimen type represents an additional source of bias, with fresh-frozen tissue, formalin-fixed paraffin-embedded samples, and surface swabs differing substantially in DNA quality, viral load, and susceptibility to contamination [22,25]. Population characteristics—including immunosuppression, geographic region, age distribution, sexual behavior, and cumulative UV exposure—further modulate both HPV prevalence and its biological relevance [11,25,26]. Finally, given the ubiquity of cutaneous HPV in normal skin, rigorous contamination controls are essential to avoid false-positive results and erroneous attribution of viral causality [11].

## 8. Clinical Implications

From a diagnostic and clinical standpoint, this anatomical gradient carries important and site-specific implications. In anogenital BD, HPV status is of central relevance, as p16 immunohistochemistry and viral testing are routinely incorporated into diagnostic algorithms, inform classification into HPV-related and HPV-independent pathways, and guide surveillance strategies and preventive interventions, including prophylactic vaccination [13,27,28,29,31,54,55]. In this setting, viral biomarkers have established prognostic and translational value and represent a cornerstone of contemporary management [7,29,54,55].

In contrast, in general cutaneous BD, the absence of a consistent causal relationship between HPV detection and tumor biology limits the clinical utility of routine viral testing. In these lesions, HPV status does not reliably correlate with histologic subtype, clinical phenotype, risk of progression, or therapeutic response, and UV-driven carcinogenesis and field cancerization remain the dominant pathogenic mechanisms [11,25,26]. Accordingly, HPV testing in extragenital cutaneous BD currently remains primarily of pathogenetic interest rather than of direct clinical relevance [11,25,26].

Nail apparatus BD represents a distinct intermediate category in which HPV detection may have emerging diagnostic and translational implications. The frequent clinical mimicry of benign nail disorders and the high prevalence of transforming α-HPV genotypes in ungual lesions suggest that awareness of HPV-associated disease may contribute to earlier recognition of malignant or premalignant nail tumors, particularly in patients with periungual verrucous lesions, longitudinal melanonychia, multifocal digital involvement, or a history of anogenital HPV infection [11,21,22,36,37].

From a therapeutic and preventive perspective, the implications of HPV involvement similarly diverge by anatomical site. Whereas HPV vaccination and viral biomarkers are already integrated into the management and prevention of anogenital intraepithelial neoplasia, their role in extragenital disease remains limited [7,55]. In contrast, the viral etiology of a substantial subset of nail unit BD has led to the hypothesis that targeted immunomodulatory therapies could be explored and prophylactic or adjuvant HPV vaccination in selected patients with recurrent, multifocal, or early-onset disease, although prospective evidence supporting these strategies remains lacking [21,36]. Collectively, these observations emphasize that HPV testing and HPV-directed interventions in BD should be anatomically contextualized, with clear clinical utility in anogenital lesions, emerging diagnostic relevance in nail apparatus disease, and limited current value in general cutaneous lesions.

A comparative overview of site-specific HPV associations and their diagnostic and clinical implications is provided in Table 3.

## 9. Conclusions

HPV prevalence in BD follows a pronounced anatomical gradient, with the strongest association in nail apparatus lesions, intermediate and inconsistent detection in general cutaneous disease, and near-universal involvement in anogenital BD. These findings support fundamentally distinct pathogenetic pathways across anatomical compartments and challenge the notion of BD as a uniform etiologic entity.

Recognition of this heterogeneity has direct implications for diagnosis, translational research, and emerging therapeutic strategies. Future investigations should focus on standardized viral detection methods, mechanistic studies of site-dependent viral oncogenesis, and interventional trials evaluating HPV-targeted therapies in selected subsets of BD.

## Figures and Tables

**Table 3:** Site-specific differences in the biological relevance of HPV in BD. The table summarizes the predominant carcinogenic drivers, relative strength of HPV involvement, genotype distribution, diagnostic utility of p16 immunohistochemistry, and clinical relevance of HPV testing across anatomical compartments.

Anatomical Site	Predominant Carcinogenic Drivers	Strength of HPV Involvement	Predominant HPV Genera/Genotypes	P16 Utility	Clinical Relevance of HPV Testing
General cutaneous (non-nail)	UV radiation, field cancerization, chronic carcinogenic exposures	Low/inconsistent	Predominantly β-HPV; occasional α-HPV	Limited, unreliable	Primarily pathogenetic interest; not recommended routinely
Nail apparatus	High-risk HPV infection facilitated by local microtrauma and genitodigital transmission	Intermediate to high	α-HPV (HPV16 most common; also 18, 31, 33, 52, 58, 73)	Variable; supportive but not definitive	Emerging diagnostic relevance in selected cases (atypical, recurrent, therapy-resistant lesions)
Anogenital region	Persistent high-risk HPV infection	High (causal)	α-HPV (HPV16, HPV18)	High; reliable surrogate marker	Central diagnostic, prognostic, and preventive relevance

## Data Availability

No new data were created or analyzed in this study. Data sharing is not applicable to this article.

## Abbreviations

The following abbreviations are used in this manuscript:

BD	Bowen disease
UV	Ultraviolet
HPV	Human papillomavirus
(pRb)	Retinoblastoma protein
EQ	Erythroplasia of Queyrat
VIN	Vulvar intraepithelial neoplasia
PeIN	Penile intraepithelial neoplasia
SCC	Squamous cell carcinoma
